# miR-21 promotes EGF-induced pancreatic cancer cell proliferation by targeting Spry2

**DOI:** 10.1038/s41419-018-1182-9

**Published:** 2018-11-21

**Authors:** Qiuyan Zhao, Sumin Chen, Zhonglin Zhu, Lanting Yu, Yingchun Ren, Mingjie Jiang, Junyong Weng, Baiwen Li

**Affiliations:** 1 Department of Gastroenterology, Shanghai General Hospital, Shanghai Jiaotong University School of Medicine, Shanghai, 201620 China; 2 Shanghai Key Laboratory of Pancreatic Diseases, Shanghai General Hospital, Shanghai Jiaotong University School of Medicine, Shanghai, 201620 China; 3 Department of General Surgery, Henan Provincial People’s Hospital, Henan, 450003 China; 4 Department of General Surgery, Shanghai General Hospital, Shanghai Jiaotong University School of Medicine, Shanghai, 201620 China

## Abstract

Pancreatic ductal adenocarcinoma (PDAC) is a highly malignant cancer that lacks effective targets for therapy. Alteration of epidermal growth factor (EGF) expression has been recognized as an essential molecular event in pancreatic carcinogenesis. Accumulating studies have demonstrated that miRNAs play critical roles in EGF signaling regulation, tumor initiation, cell proliferation and apoptosis. Here, we demonstrated that miR-21 expression was induced by EGF in pancreatic cancer cells. miR-21 promoted EGF-induced proliferation, inhibited cell apoptosis and accelerated cell cycle progression. In vivo experiments confirmed the influence of miR-21 on tumor growth. Mechanistic studies revealed that miR-21 targeted MAPK/ERK and PI3K/AKT signaling pathways to modulate cell proliferation. In addition, Spry2 was proven to be a target of miR-21. Furthermore, miR-21 and Spry2 were significantly related to clinical features and may be valuable predictors of PDAC patient prognosis.

## Introduction

Pancreatic ductal adenocarcinoma (PDAC) is highlighted by poor prognosis, and PDAC-associated mortality closely parallels incidence^1^. Due to lack of effective modalities for early detection, most PDAC patients are in the late stages of disease and not candidates for surgical resection. Worldwide, more than 200,000 people die from pancreatic cancer every year^2^. Total deaths from pancreatic cancer have increased dramatically, and pancreatic cancer is predicted to become the second leading cause of cancer-related deaths by 2030^3^. Therefore, new insight into the underlying molecular pathophysiology of PDAC is urgently needed to advance the development of early detection strategies and effective therapeutic targets.

At the molecular level, pancreatic cancer exhibits high frequency of genetic alterations, including KRAS, TP53, CDKN2A and SMAD4 alterations, and aberrant activation of mitogenic signaling pathways as a consequence of overexpression of receptor tyrosine kinase (RTKs), such as epidermal growth factor (EGF) receptor (EGFR) and its ligands^4^.  Elevated EGFR expression is detected during tumor progression from early pancreatic intraepithelial neoplasia to PDAC and has been recognized as the essential molecular alteration in pancreatic carcinogenesis^4^. EGF activates the RAF-mitogen-activated protein kinase (MAPK) and phosphoinositide-3-kinase (PI3K) pathways, which leads to enhanced cell proliferation and survival^5^. However, the potential molecular mechanisms leading to constitutive activation of these pathways have not been fully elucidated. Particularly, it is important to identify the regulators of these pathways in PDAC.

MicroRNAs (miRNAs) are small endogenous noncoding RNAs that exert their negative regulatory functions via mRNA degradation or translational inhibition^6–8^. Through interactions with the 3′ untranslated region (3′ UTR) of mRNAs, miRNAs can regulate the expression of many genes and modulate a broad range of cellular signaling pathways, among which pathways driving tumorigenesis are of particular importance^9^. Increasing evidences have indicated that miRNAs dysregulation is involved in tumor initiation, cell proliferation, apoptosis, angiogenesis, and metastasis^8,10,11^. For example, miR-96 can decrease pancreatic cancer cell proliferation, migration, and invasion by suppressing the expression of KRAS^12^. microRNA-182, which suppresses SMAD7 protein, promotes TGFß-induced cancer cell invasion and metastasis^13^. In hepatocellular carcinoma (HCC), miR-1207-5p inhibits HCC cell growth and invasion by suppressing the AKT/mTOR signaling pathway through fatty acid synthase inhibition^14^.

Although both EGFR signaling and miRNAs can profoundly influence pancreatic cancer cell behavior, the role of miRNAs in EGF-mediated phenotypes is poorly defined. Studies have demonstrated that EGF can induce differential expression of miRNAs which then targeted a group of mRNAs regulating the activity of signal pathways^15^. Thus, growth factor-inducible changes in the levels of miRNAs and mRNAs may create a feedback regulatory system, which is often defective in the tumor formation process.

In this study, we demonstrate that EGF can induce the expression of miR-21, which enhances EGF-induced pancreatic cancer cell survival by targeting the MAPK/ERK and PI3K/AKT signaling pathways. Then, Sprouty2 (Spry2) is identified as the target of miR-21 and found to mediate the function of miR-21 in PDAC cells. Furthermore, we show that miR-21 and Spry2 are correlated with pancreatic cancer clinical pathological features. Our results reveal a novel mechanism to disengage the negative feedback of EGF signal pathways during pancreatic cancer cell proliferation.

## Materials and methods

### Patient tissue samples and cell lines

PDAC tumors and their adjacent pancreatic normal tissues were collected from Shanghai General Hospital. None of the patients had received radiotherapy or chemotherapy before surgery. Written informed consent for research purposes was obtained before enrollment in the research project. This study was approved by the Ethics Committee of Shanghai General Hospital of Shanghai Jiaotong University.

The human pancreatic cancer cell lines PANC-1, MIA PaCa-2, CFPAC-1 and normal pancreatic ductal epithelial cells (HPDE6-c7) were cultured in DMEM (Gibco) supplemented with 10% fetal bovine serum (FBS; Gibco). SW-1990 and AsPC-1 cells were cultured in RPMI-1640 medium (HyClone) with 10% FBS (Gibco). All of the cells were cultured at 37 °C with 5% CO2.

### Tissue microarray (TMA)

The clinical significance of miR-21 and Spry2 expression in PDAC patients was analyzed using TMAs purchased from Shanghai Outdo Biotech (Shanghai, China) that contained 63 pancreatic cancer tissues and adjacent normal pancreatic tissues. Shanghai Outdo Biotech also provided patients’ information including sex, age, overall survival, clinical stage, and lymph node metastasis. Use of the tissue microarrays complied with relevant regulations, and was approved by the Ethics Committee of Shanghai General Hospital.

### RNA extraction and quantitative real-time PCR (qRT-PCR)

Total RNA from PDAC tissues and cells was extracted using RNAiso Plus Reagent (TakaRa) according to the manufacturer’s protocol. 500 ng of RNA was reverse-transcribed with PrimeScript™ RT reagent kit (TakaRa). Then, cDNA was amplified with SYBR® Premix Ex Taq™ (TakaRa). The gene expression levels were determined by the ΔΔCt method with U6 and GAPDH as internal controls for miRNA and mRNA respectively.

### Western blotting analysis

Protein samples were lysed in RIPA buffer supplemented with protease and phosphatase inhibitors (Roche), and the concentration of protein was measured with BCA Protein Assay Kit (Tiangen, Beijing, China). Protein lysates were separated by SDS-PAGE and then transferred onto PVDF membranes (Millipore, Billerica, MA, USA). The membranes were blocked in 5% fat-free milk at room temperature for 1.5 h and incubated with specific primary antibodies at 4 °C overnight. Subsequently, the membranes were incubated with HRP-conjugated secondary antibody for 2 h at room temperature. Detection was performed using ECL reagent (Millipore, MA, USA).

### Transient transfection

The miR-21 mimics, mimics negative control, miR-21 inhibitor and inhibitor negative control oligonucleotides were purchased from GenePharma (Shanghai, China). The Spry2 overexpression plasmid and corresponding empty vector were constructed by GenePharma (Shanghai, China). Specific siRNA against Spry2 and a scramble siRNA were synthesized by RiboBio (Guangzhou, China). Pancreatic cancer cells cultured to 60-70% confluence were transfected with the above oligonucleotides and vectors using Lipofectamine™2000 in accordance with the manufacturer’s instructions (Invitrogen).

### Cell counting and cell proliferation assay

Cells were seeded into 96-well plates with 3000 cells per well and cultured at 37 °C in a humidified atmosphere with 5% CO2. Cell counts were determined using Celigo® Image Cytometer (Nexcelom, USA) after the cells were incubated for 36 h.

For the CCK8 proliferation assay, 3000 cells/well were cultured under the same conditions described above. After 24 h, 10 μl/well of CCK-8 (Dojindo, Tokyo Japan) was added into the 96-well plates. Then, the absorbance (OD value) was measured at 450 nm 2 h later. CCK8 assay was performed at the indicated time point of everyday for 5 days. All experiments were executed in triplicate.

For the EdU (RiboBio, Guangzhou, China) cell proliferation assay, cells were plated into 24-well plates and cultured for 24 h. Then, the cells were incubated with 50μM EdU for 2 h before fixation, permeabilization and EdU staining. Cell nuclei were stained with Hoechst 33342 for 30 min. Images were captured using a fluorescence microscope (Olympus, Tokyo, Japan). The number of EdU-positive cells was calculated as follows: (EdU add-in cells/Hoechst stained cells)*100%. All experiments were executed in triplicate.

### Flow cytometric analysis

The cell apoptosis assay was performed using the Annexin V-FITC/Propidium Iodidie (PI) Apoptosis Detection Kit (eBioscience, 88-8005, USA) according to the manufacturer’s instructions. Briefly, cells were suspended in 100 μl of binding buffer, and then, 5 μl of fluorochrome-conjugated Annexin V was added. After incubation for 15 min at room temperature, the cells were resuspended in 200 μl of binding buffer and 5 μl of PI was added. Then, the apoptotic cells were detected and analyzed by flow cytometry.

For the cell cycle assay, pancreatic cancer cells were collected and fixed in 70% ethanol overnight. After being washed in precooled PBS, the cells were stained with 10 μl of RNase A and 25 μl of PI in 500 μl of dye buffer at room temperature for 30 min.

### Lentiviral vector construction and transfection

The miR-21 overexpressing and suppressing lentiviruses and their respective negative controls were constructed by Hanbio (Shanghai, China). PANC-1 cells and MIA PaCa-2 cells were cultured in 6-well plates. When the cells reached 50% confluence, they were infected with the appropriate lentiviruses in the presence of 6μg/ml polybrene (Hanbio, Shanghai, China). Stable cell lines were selected by using 4μg/ ml puromycin (Sigma, USA) for 2 weeks (Tables. 1 and 2).

**Table 1:** Correlations between miR-21 and clinicopathologic parameters in pancreatic cancer patients

Parameters	No.	miR-21 expression		*χ* ^2^	*P*
	(*n* = 63)	high (*n* = 47)	low (*n* = 16)		
*Gender*					
Male	36	24	12	1.901	0.168
Female	27	23	4		
*Age (years)*					
≤60	24	19	5	0.426	0.514
>60	39	28	11		
*Tumor location*					
Head	42	30	12	0.262	0.609
Body/tail	21	17	4		
*Tumor size (cm)*					
≤3	14	13	1	2.048	0.152
>3	49	34	15		
*Pathologic grade*					
I-II	41	28	13	1.606	0.205
III	22	19	3		
*T stage*					
T1-T2	22	13	9		
T3*	41	34	7	4.293	**0.038**
*N stage*					
N0*	37	23	14	5.819	**0.016**
N1	26	24	2		
*AJCC stage*					
0-IIA**	36	21	15	9.818	**0.002**
IIB-IV	27	26	1		
Perineural invasion				1.901	0.168
Absent	27	23	4		
Present	36	24	12		
Vascular invasion				0.655	0.419
Absent	48	37	11		
Present	15	10	5		

*P <0.05; **P < 0.01

*P <0.05; **P < 0.01

**Table 2:** Correlations between Spry2 expression and clinicopathologic parameters of pancreatic cancer patients

Parameters	No.	Spry2 expression		*χ* ^2^	*P*
	(*n* = 63)	high (*n* = 21)	low (*n* = 42)		
*Gender*				2.624	0.105
Male	36	15	21		
Female	27	6	21		
*Age (years)*					
≤60	24	8	16	0.000	1.000
>60	39	13	26		
*Tumor location*					
Head	42	12	30	1.286	0.257
Body/tail	21	9	12		
*Tumor size (cm)*				0.394	0.530
≤3	15	6	9		
>3	48	15	33		
*Pathologic grade*					
I-II*	41	18	23	4.618	**0.032**
III	22	3	19		
*T stage*					
T1-T2*	22	11	11	4.226	**0.040**
T3	41	10	31		
*N stage*					
N0*	37	17	20	5.116	**0.024**
N1	26	4	22		
*AJCC stage*					
0-IIA**	36	17	19	7.292	**0.007**
IIB-IV	27	4	23		
Perineural invasion				1.167	0.280
Absent	27	7	20		
Present	36	14	22		
Vascular invasion				0.459	0.498
Absent	48	15	34		
Present	15	6	9		

*P <0.05; **P < 0.01

### Luciferase reporter assay

PANC-1 cells and MIA PaCa-2 cells were seeded in 24-well plates at approximately 1 × 10^5^/well the day before transfection. Cells were cotransfected with approximately 100 ng of wild-type or mutant psiCHECK2-hSpry2-3'UTR and with miR-21 mimics (5 nM) or negative control (50 nM) and miR-21 inhibitor (100 nM) or negative control (100 nM) using Lipofectamine™2000 (Invitrogen). At 36 h after transfection, luciferase activities were measured using the Dual Luciferase Reporter Assay System (Promega). All experiments were executed in triplicate.

### Immunohistochemistry (IHC)

The TMA was deparaffinized in xylene and dehydrated through a graded alcohol series. Then, antigen retrieval was performed by boiling the TMA in sodium citrate solution (0.01 M, pH 6.0) for 15 min. After blocking endogenous peroxidase activity using 3% hydrogen peroxide, the TMA was incubated with Spry2 antibody (1:50 Abcam, Cambridge,UK) at 4 °C overnight, and then incubated with secondary antibody for 1 h at room temperature.

### In situ hybridization (ISH)

The level of miR-21 in the TMA was evaluated by ISH using a specific digoxin-labeled miR-21 probe. After deparaffination, the TMA was treated with proteinase K at 37 °C for 10 min and then with hybridization mix for 1 h at 57 °C. After being blocked in blocking solution for 15 min, the TMA was incubated with digoxin-labeled miR-21 probe at 50 °C overnight. Then, the TMA was hybridized with the anti-digoxin and horse radish peroxidase at room temperature for 2 h. After the TMA was washed and dehydrated, it was mounted with coverslips.

The staining scores were based on staining intensity and the proportion of stained cells. Staining intensity was scored as 0 (negative staining), 1 (weak staining), 2 (moderate staining), or 3 (strong staining). The proportion of stained cells was scored as follows: 0 (0–10%), 1 (10–25%), 2 (25–50%), 3 (50–75%), or 4 (75–100%). The total score of each section was acquired by multiplying the above two scores. For statistical analyses, the tissues were divided into two groups based on the score: total scores of ≤4 and >4 were defined as low and high expression, respectively.

### Animal experiments

To study primary tumor growth, 4-week-old male BALB/c nude mice were randomly divided into 4 groups (*n* = 5), and 1.0 × 10^6^ cells from stable cells lines were subcutaneously injected into the right flank of the nude mice. Tumor size was monitored twice a week, and tumor volume was estimated using the following formula: volume = width^2^ × length × π/6. There weeks later, all the mice were anesthetized and administered 15 mg/kg D-luciferin (Promega, USA) through intraperitoneal injection; tumor size was imaged by the Xenogen IVIS Illumina System (Caliper Life Sciences, USA). All the mice were sacrificed 3 weeks’ postinjection, and xenografts were removed and weighed. All experiments involving animals in this study were approved by the Institutional Animal Care and Use Committee of Shanghai General Hospital.

### Statistical analysis

SPSS 22.0 was used for statistical analysis. Significant correlations between miR-21/Spry2 expression and clinicopathological features of PDAC patients were determined by Wilcoxon rank sum test. Survival difference was evaluated using the Kaplan–Meier method and a log-rank test. *P* < 0.05 was considered statistically significant for all tests

## Results

### miR-21 is required for EGF-induced pancreatic cancer cell survival in vitro

**Fig. 1:**
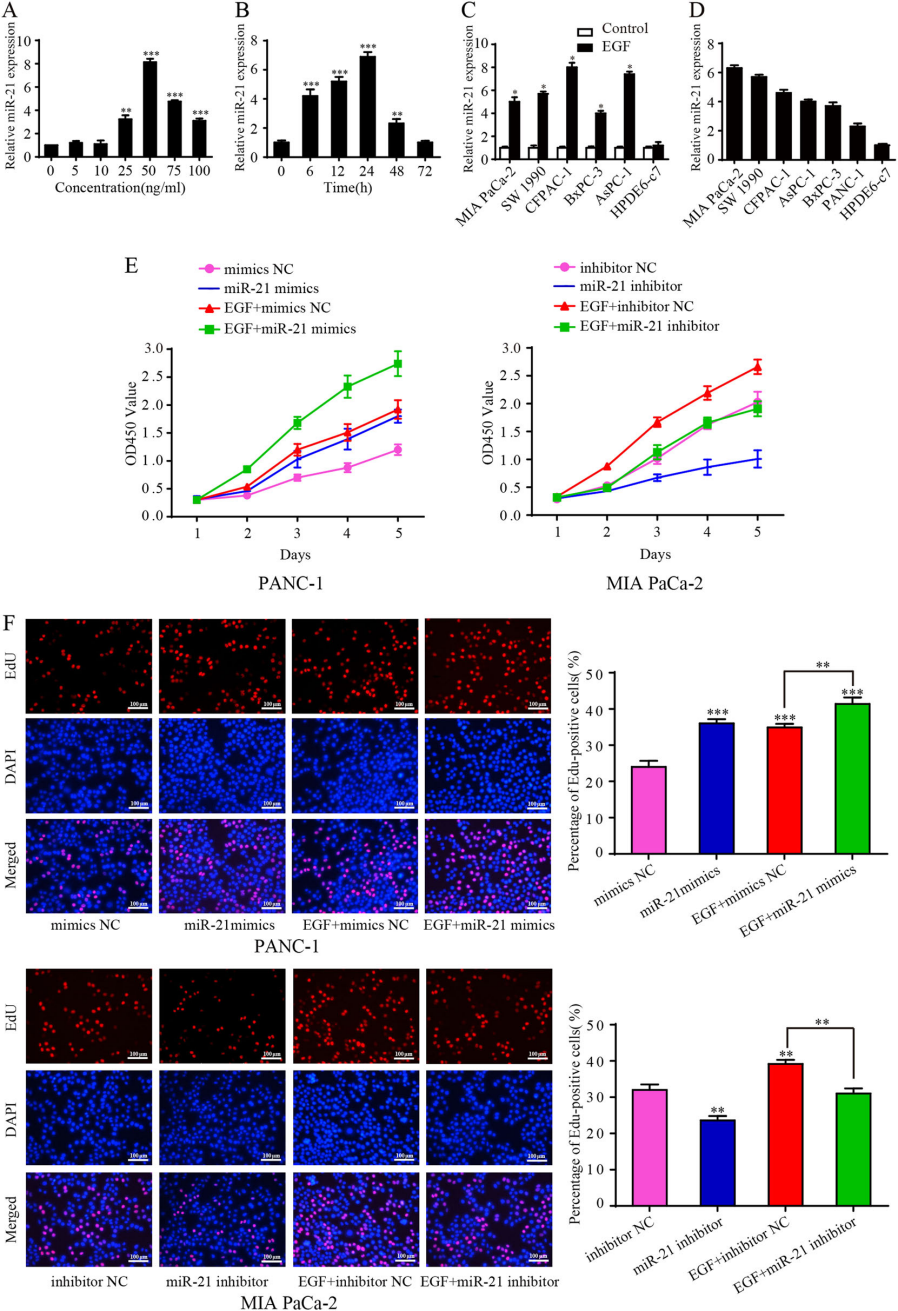
miR-21 is induced by EGF and is required for EGF-induced pancreatic cancer cell proliferation. **a** The concentration-dependent effect of EGF on miR-21 level in PANC-1 cells. **b** The time-dependent effect of EGF on miR-21 level in PANC-1 cells. **c** miR-21 expression in other cell lines after 24 h of EGF treatment. **d** Relative expression of miR-21 in pancreatic cancer cell lines compared with normal pancreatic ductal epithelial cells. **e** After being serum-starved for 24 h, PANC-1 and MIA PaCa-2 cells were transfected with miR-21 mimics and inhibitor or negative control and simultaneously treated with or without EGF (50 ng/ml). CCK8 proliferation was performed to examine the growth of PANC-1 cells and MIA PaCa-2 cells. **f** EdU assay was used to further analyze the proliferative activity of PANC-1 cells and MIA PaCa-2 cells. Three independent experiments were performed for each group. All data are shown as the mean ± SD. *P <0.05; **P < 0.01; ***P < 0.001

Previously, it was demonstrated that miR-21 expression could be induced by overexpression of KRAS and TGF-ß. Meanwhile, Erbitux, an EGFR inhibitor, significantly reduced miR-21 levels^16^. Therefore, we further assessed whether miR-21 could be induced by EGF in PANC-1cells. We observed that EGF had no effect on miR-21 level at the concentration of 5 and 10 ng/ml, while EGF at the concentration of 25 to 75 ng/ml increased the expression of miR-21 and its expression level reached a peak value at 50 ng/ml (Fig. 1a). Then, PANC-1 cells were also treated with 50 ng/ml EGF for 6 to 72 h. We found that miR-21 level was increased in a time-dependent manner and reached a peak value at 24 h (Fig. 1b). Overexpression of miR-21 under EGF stimulation was also confirmed in other pancreatic cancer cell lines (Supplementary Figure S1 and Fig. 1c). However, EGF treatment did not upregulate miR-21 transcript in normal pancreatic ductal epithelial cell line HPDE6-c7 (not shown). In order to detect miR-21 expression in PDAC cell lines, qRT-PCR was implemented. miR-21 was confirmed to be relatively higher in PDAC cell lines than in HPDE6-c7 cells (Fig. 1d). Among these PDAC cell lines, PANC-1 cells, showing the lowest miR-21 expression, and MIA PaCa-2 cells, showing the highest miR-21 expression, were selected for the following experiments.

To investigate the role of miR-21 in EGF-induced proliferation, PANC-1 cells were transfected with miR-21 mimics or NC (negative control) and simultaneously stimulated with 50 ng/mL EGF. CCK-8 proliferation and EdU assays indicated that both miR-21 and EGF could facilitate cell proliferation; moreover, EGF-stimulated cell proliferation was enhanced by miR-21 (Fig. 1e, f). In contrast, miR-21 inhibitor suppressed EGF-mediated cell proliferation in MIA PaCa-2 cells (Figs. 1e, f).

### miR-21 promotes cell cycle progression and inhibits cell apoptosis

**Fig. 2:**
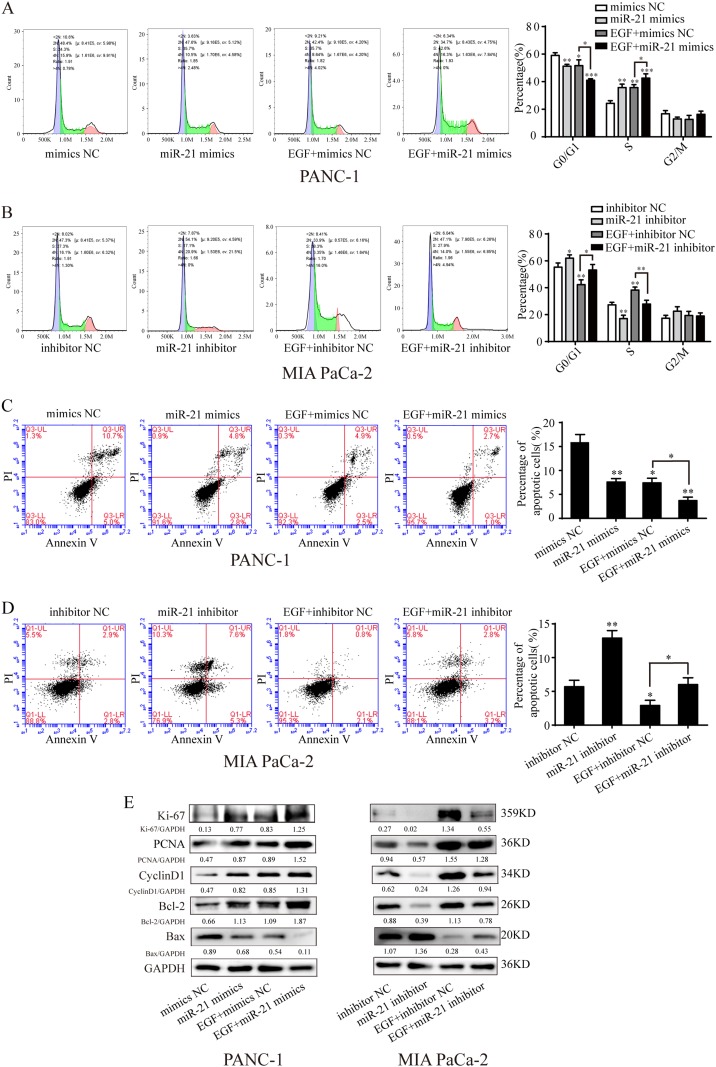
miR-21 overexpression decreases the percentage of cells in G1 phase and inhibits cell apoptosis. **a**, **b** After being serum-starved for 24 h, PANC-1 and MIA PaCa-2 cells were transfected with miR-21 mimics and inhibitor or negative control and simultaneously treated with or without EGF (50 ng/ml). Flow cytometry analysis of cell cycle of PANC-1 cells and MIA PaCa-2 cells. **c**, **d** After being serum-starved for 24 h, PANC-1 and MIA PaCa-2 cells were transfected with miR-21 mimics and inhibitor or negative control and simultaneously treated with or without EGF (50 ng/ml). Flow cytometry analysis of cell apoptosis in PANC-1 cells and MIA PaCa-2 cells. **e** After being serum-starved for 24 h, PANC-1 and MIA PaCa-2 cells were transfected with miR-21 mimics and inhibitor or negative control and simultaneously treated with or without EGF (50 ng/ml) for 48 h, expression of proliferation marker, cell cycle-related protein and apoptosis-related proteins in PANC-1 and MIA PaCa-2 cells. Western blotting expression densitometry data was quantified by imageJ and then was normalized to the corresponding densitometry of GAPDH. Three independent experiments were performed for each group. All data are represented as the mean ± SD. * *P* < 0.05; **P < 0.01; ***P < 0.001

To explore how miR-21 regulates EGF-induced cell proliferation, we investigated the effects of miR-21 on cell cycle distribution and cell apoptosis. Both miR-21 and EGF treatment decreased the distribution of PANC-1 cells in G0/G1 phase (Fig. 2a). miR-21 overexpression further promoted the transition from G0/G1 phase to S phase under EGF stimulation (Fig. 2a). Meanwhile, decreased cell numbers in the G0/G1 phase under EGF treatment could be abrogated by the suppression of miR-21 (Fig. 2b). As expected, miR-21 also had a significant influence on pancreatic cancer cell apoptosis, as determined by Annexin V apoptosis assay. In PANC-1 cells, the combined effect of miR-21 and EGF on cell apoptosis was significantly greater than the effect of either miR-21 or EGF alone. Few apoptotic cells were detected in miR-21 overexpressing PANC-1 cells under treatment with EGF (Fig. 2c), whereas miR-21 inhibition increased the percentage of apoptotic cells in MIA PaCa-2 cells (Fig. 2d). To better understand the mechanism by which miR-21 regulates cell proliferation, cell cycle progression and cell apoptosis, we examined the expression of several key regulars in these processes. Western blotting analyses showed that under EGF stimulation, overexpression of miR-21 remarkably increased the expression of proliferation markers Ki-67 and PCNA. Moreover, higher level of the cell cycle-related protein CyclinD-1 was observed in PANC-1 cells. Finally, the anti-apoptotic protein Bcl-2 was elevated while pro-apoptotic protein Bax was decreased with enhanced miR-21 expression (Fig. 2e). In contrast, inhibition of miR-21 decreased the levels of Ki-67, PCNA, CyclinD-1 and Bcl-2, and promoted Bax expression in MIA PaCa-2 cells (Fig. 2e). These results support a role of miR-21 in inducing G1/S transition and inhibiting cell apoptosis.

### miR-21 promotes pancreatic cancer growth in vivo

**Fig. 3:**
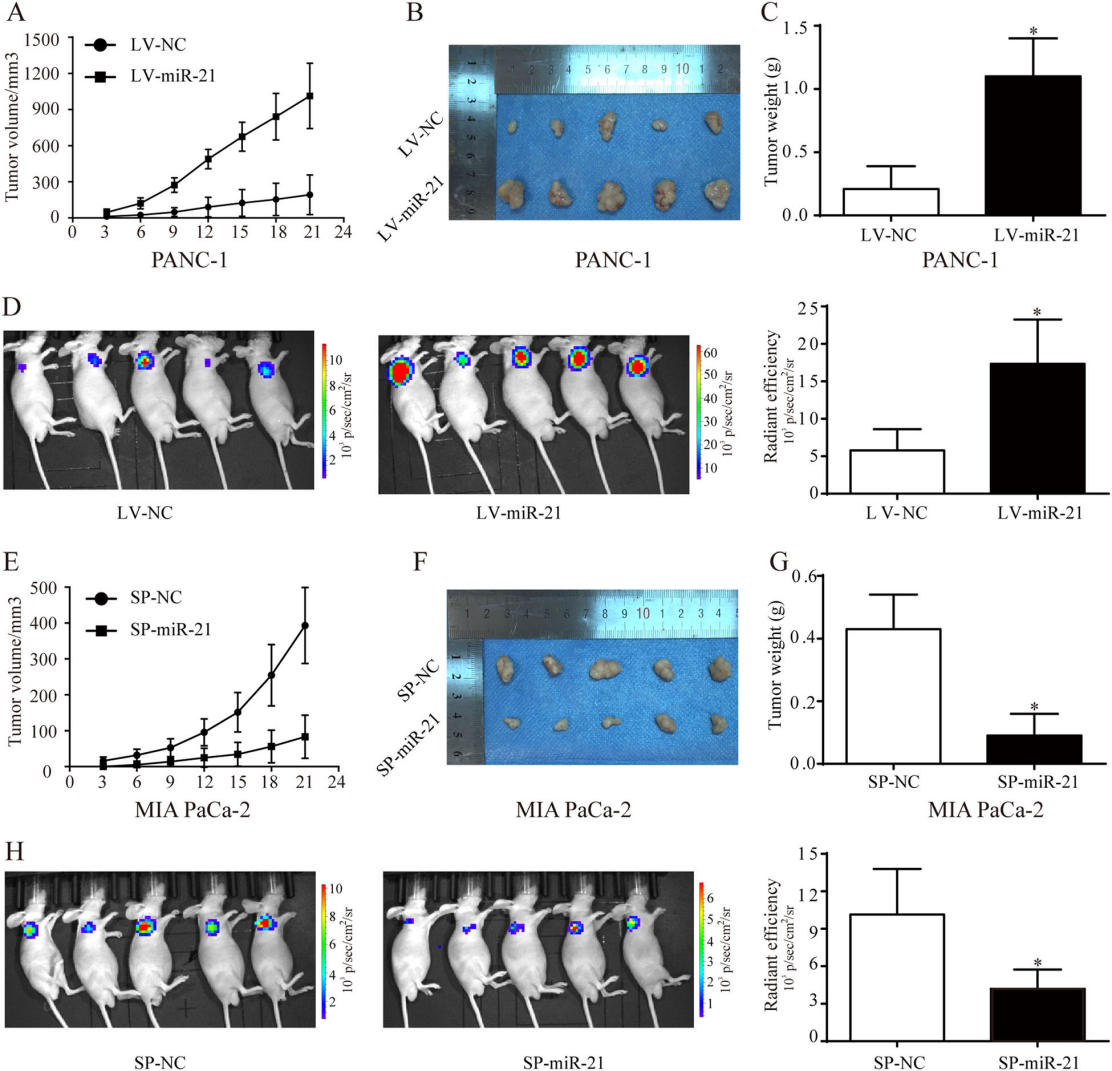
miR-21 promotes pancreatic cancer growth in vivo. **a**, **e** The graph shows the growth of tumors in each group. **b**, **c** The tumors were removed 21 days after LV-NC cells and LV-miR-21 cells were subcutaneously injected, and tumor weights were measured. **d**, **h** Bioluminescence imaging exhibits the final tumor volume of each group. **f**, **g** The tumors were removed 21 days after SP-NC cells and SP-miR-21 cells were subcutaneously injected and tumor weights were measured. All data are shown as the mean ± SD. *P < 0.05

To further verify the role of miR-21 in pancreatic cancer growth in vivo, the nude mouse xenograft models of pancreatic cancer were constructed. PANC-1 cells were transfected with lentiviral vectors of miR-21 overexpression or negative control to construct two stable cell lines: LV-miR-21/PANC-1 and LV-NC/PANC-1, and MIA PaCa-2 cells were transfected with lentiviral vectors of miR-21 sponge or negative control to construct two stable cell lines: SP-miR-21/MIA PaCa-2 and SP-NC/MIA PaCa-2. The stable cell lines LV-NC, LV-miR-21 and SP-NC, SP-miR-21 were subcutaneously injected into the nude mice. The tumor volume was monitored twice a week. We found that the tumors derived from LV-miR-21 cells grew remarkably faster than those derived from LV-NC cells (Fig. 3a). Three weeks after cell injection, the nude mice were euthanized, and the tumors were removed (Fig. 3b). The final tumor weight was much higher in the LV-miR-21 group than in the LV-NC group (Fig. 3c). Consistently, bioluminescence imaging (BLI) showed that LV-miR-21 mice exhibited significantly higher luciferase activity than LV-NC mice (Fig. 3d). Similar results were confirmed in SP-NC and SP-miR-21 xenograft models. Tumor volume and weight were lower in the SP-miR-21 mice than in the SP-NC mice (Fig. 3e, f,m, g). Meanwhile, the luciferase activity of SP-miR-21 tumors was weaker than that of SP-NC tumors (Fig. 3h). Collectively, our data demonstrated a functional role for miR-21 in promoting pancreatic tumor growth in vivo.

### miR-21 affects pancreatic cancer cell proliferation by targeting the MAPK/ERK and PI3K/AKT signaling pathways

**Fig. 4:**
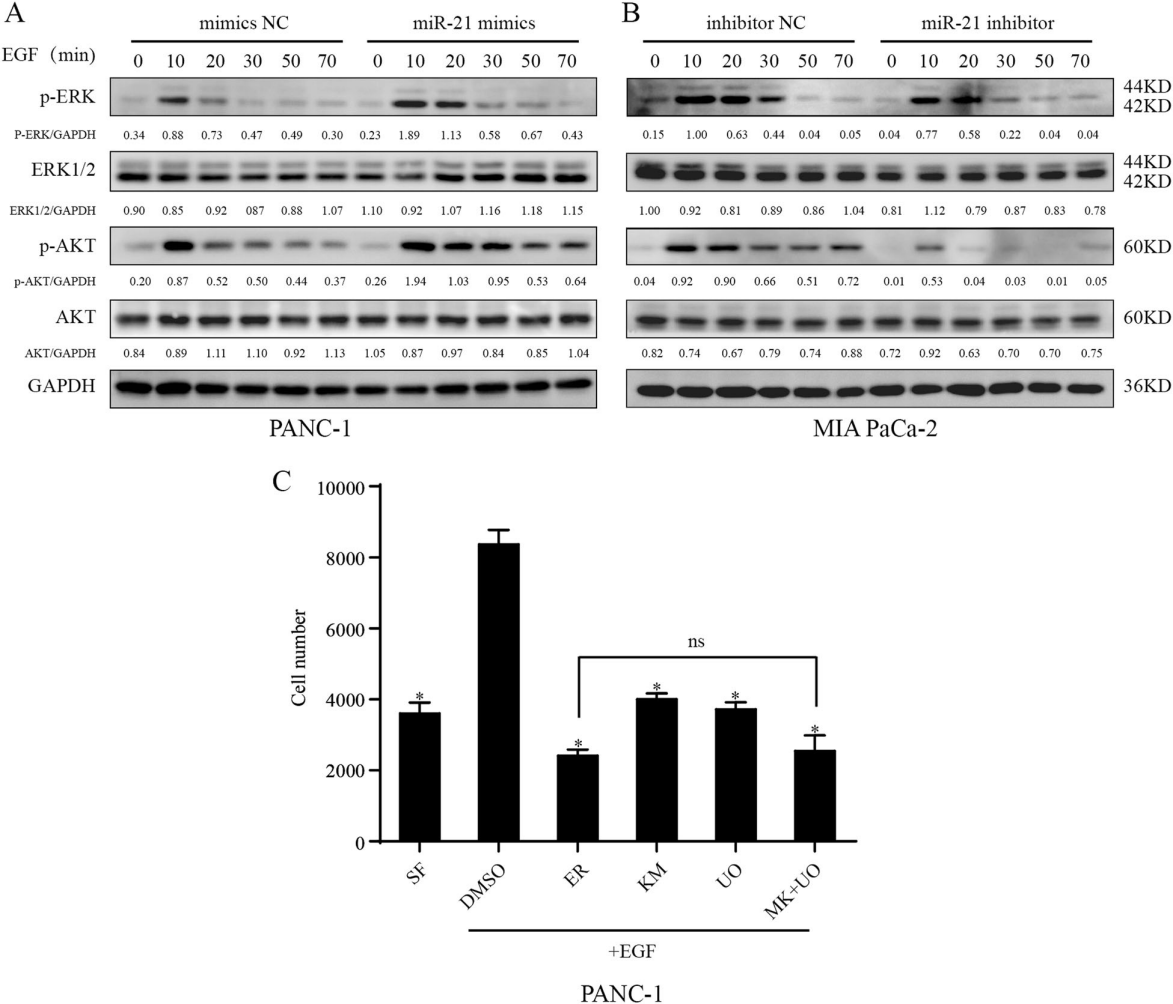
Effects of miR-21 on EGF signaling and effects of signaling inhibitors on EGF-induced cell growth. **a**, **b** After being serum-starved for 24 h, PANC-1 and MIA PaCa-2 cells were transfected with miR-21 mimics and inhibitor or negative control and simultaneously treated with or without EGF (50 ng/ml) for the indicated times. Western blotting analysis of the indicated proteins was then carried out. Western blotting expression densitometry data was quantified by imageJ and then was normalized to the corresponding densitometry of GAPDH. **c** After being serum-starved for 24 h, PANC-1 cells transfected with miR-21 mimics were incubated for 36 h without EGF (SF) or with EGF(50 ng/ml) in the absence (DMSO) or presence of erlotinib (1 µM), MK-2206 (1 µM) or UO126 (1 µM) and the cell numbers were counted. Three independent experiments were performed for each group. All data are represented as the mean ± SD. *P < 0.05 compared with DMSO group. ns, not significant between the ER and MK + UO groups

Overexpression of EGFR, a cell surface protein that can be triggered by binding to its growth factor ligands, such as EGF, is associated with abnormal cell growth and malignant phenotype^17^. Our results showed that miR-21 played important roles in EGF-induced pancreatic cancer cell proliferation. To further test the role of miR-21 in these effects, we examined the activity of two major EGFR signaling pathways—the MAPK/ERK and PI3K/AKT signaling cascades by western blotting with phosphorylation-specific antibodies. EGF treatment rapidly increased ERK1/2 (Thr-202/Tyr-204) and AKT (Ser473) phosphorylation in mimics NC cells within 20 min, and miR-21 mimics dramatically enhanced the amplitude and duration of ERK1/2 and AKT phosphorylation which was still detectable after 70 min (Fig. 4a). In contrast, miR-21 inhibitor significantly reduced ERK1/2 and AKT phosphorylation (Fig. 4b). Next, special inhibitors were used to explore the effects of these two signaling pathways on pancreatic cancer cell survival in the presence of miR-21. The EGFR inhibitor erlotinib (1 µM) completely blocked EGF-induced cell proliferation in the presence of miR-21 in PANC-1 cells (Fig. 4c), while the AKT inhibitor MK-2206 (1 µM) and the MEK inhibitor UO126 (1 µM) partially blocked cell growth (Fig. 4c). Then, we found that the combination of MK-2206 and UO126 was as effective as erlotinib in suppressing EGF-mediated growth (Fig. 4c). Taken together, our findings validate that miR-21 can promote pancreatic cancer cell growth and inhibit cell apoptosis through the MAPK/ERK and PI3K/AKT signaling pathways.

### Spry2 is a direct target of miR-21 in pancreatic cancer cells

**Fig. 5:**
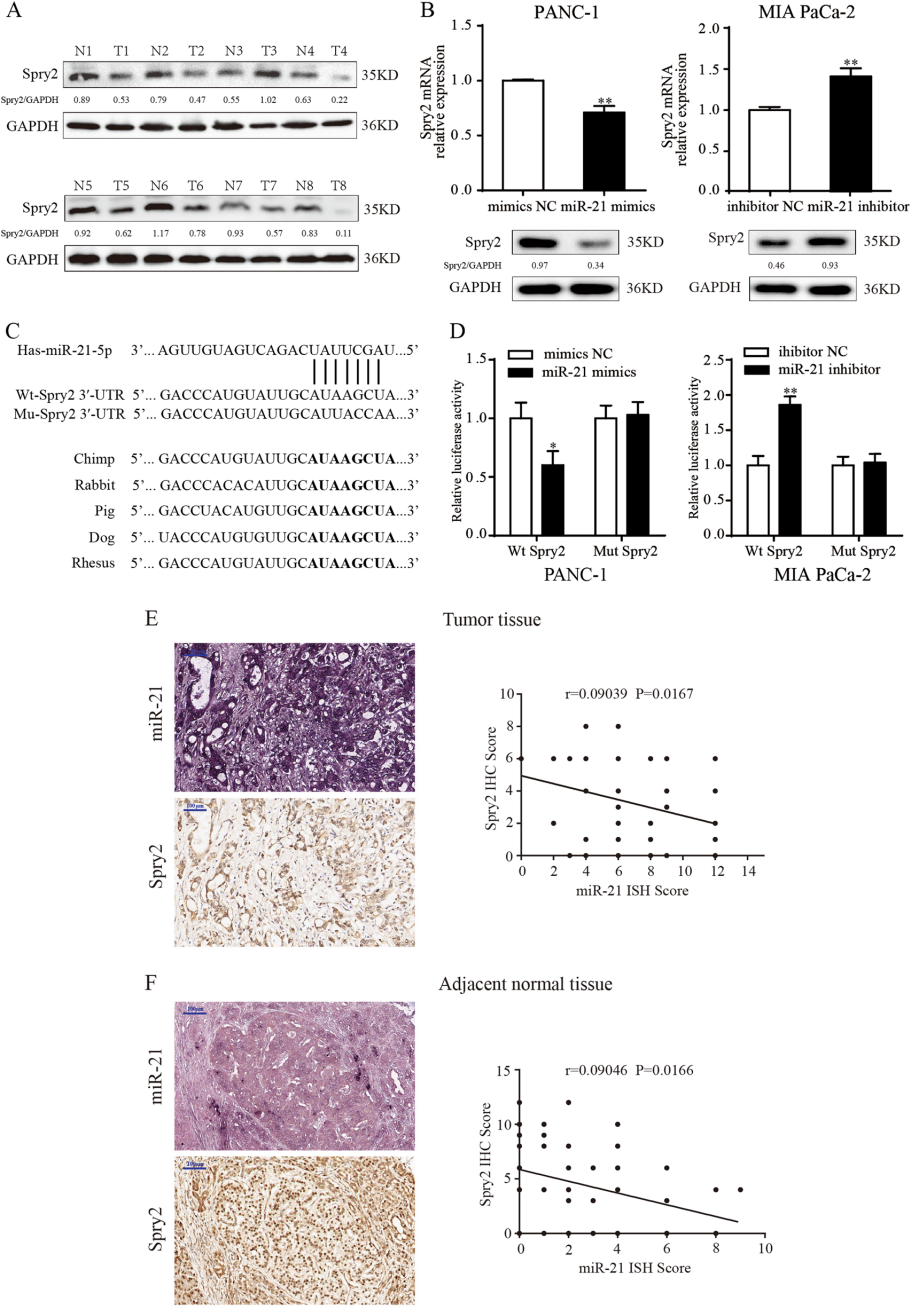
Spry2 is experimentally confirmed to be a direct target of miR-21. **a** Western blotting confirmed that Spry2 expression was downregulated in PDAC tissues compared with adjacent normal pancreatic tissues. **b** miR-21 overexpression in PANC-1 cells reduced both the mRNA and protein level of Spry2. miR-21 suppression in MIA PaCa-2 cells increased the Spry2 expression at both the mRNA and protein level). **c** miR-21 and its putative binding sequences in the human 3′-UTR of Spry2. The binding sequences in the complementary sites were replaced by the indicated nucleotides to produce mutant Spry2 3′-UTR luciferase reporter constructs. Marked sequences represent the conserved complementary nucleotides of the miR-21 binding sequence in various mammals. **d** Luciferase activity of Spry2 wild-type and mutated 3′-UTR with miR-21 mimics or inhibitor. **e**, **f** The relationship between miR-21 and Spry2 was determined by Pearson’s correlation analysis based on the ISH and IHC scores. Three independent experiments were performed for each group. All data are represented as the mean ± SD. *P < 0.05 **P < 0.01

To gain insight into the molecular mechanisms through which miR-21 exerts its functional effects in pancreatic cancer, we predicted potential targets using three different commonly used miRNA target prediction algorithms–TargetScan, miRBase and Picta. More than two hundred genes were potential targets of miR-21. Among those potential candidates, we focused on Spry2, one of the Sprouty family members which have been characterized as antagonists of RTKs signaling^18^. Several studies have demonstrated that Spry2 could inhibit growth factor-induced cell proliferation and migration^19,20^ and acted as a tumor suppressor in many types of cancer^21,22^. Public data from Oncomine showed that the expression level of Spry2 was lower in PDAC tissues than in normal tissues (Supplementary Fig. S2 A and B). To assess the expression of Spry2 in pancreatic cancer, western blotting was conducted in 8 pairs of pancreatic cancer tissues and adjacent normal pancreatic tissues (Fig. 5a). We further confirmed that Spry2 was poorly expressed in pancreatic cancer tissues.

To verify the hypothesis that Spry2 might be the target of miR-21, the abundance of Spry2 was analyzed at both the mRNA and protein levels in miR-21 mimics, miR-21 inhibitor cells and their respective negative controls. As expected, miR-21 mimics decreased, whereas miR-21 inhibitor increased the expression of Spry2 mRNA and protein in PDAC cells (Fig. 5b). Bioinformatics analyses also revealed that Spry2 3’-UTR contained one putative miR-21 binding site and the binding site was exactly conserved across species (Fig. 5c). Next, to further confirm that Spry2 is a target of miR-21, we conducted a wild-type (wt) Spry2 3’-UTR luciferase construct and a mutant construct (mt) in which 5 nucleotides in the potential binding site were replaced (Fig. 5c). The luciferase reporter assay indicated that luciferase activity was significantly decreased after co-transfection of cells with miR-21 mimics and the wild-type Spry2 3′-UTR construct for 48 h (Fig. 5d). Conversely, miR-21 inhibition increased the luciferase activity of the wild-type Spry2 3′-UTR (Fig. 5d). However, alteration of miR-21 expression showed no significant impact on the luciferase activity of the mutant Spry2 3′-UTR (Fig. 5d). More importantly, ISH and IHC scores revealed that there was a negative correlation between miR-21 and Spry2 in pancreatic cancer tissues (Figs. 5e, f). In summary, our results suggested that miR-21 directly recognized the 3′-UTR of Spry2 mRNA and regulated Spry2 expression by degrading Spry2 mRNA in pancreatic cancer cells.

### Spry2 mediates the effects of miR-21 on pancreatic cancer cells

Previous studies have demonstrated that Spry2 acts as a tumor suppressor in many other cancer types^21,22^. To corroborate the functions of Spry2 in pancreatic cancer cells, PANC-1 and MIA PaCa-2 cells were chosen for Spry2 knockdown and overexpression, respectively. The levels of Spry2 protein were confirmed by western blotting (Supplementary Fig. S 2C). In PANC-1 cells, Spry2 was knocked down by a specific siRNA (si-Spry2). CCK-8 proliferation and EdU assays demonstrated that si-Spry2 increased EGF-induced proliferation (Supplementary Fig. S2 D and E). Moreover, MIA PaCa-2 cells were transfected with empty vector (EV) or Spry2 plasmid. We found that Spry2 overexpression led to a significant reduction of cell proliferation under EGF stimulation in MIA PaCa-2 cells (Supplementary Fig. S 2D and E). Then, flow cytometric analysis was performed to examine the effect of Spry2 on pancreatic cancer cell proliferation through alteration of altering cell cycle progression and cell apoptosis. The results revealed that si-Spry2 promoted the transition from G0/G1 phase to S phase and reduced the number of apoptotic cells under EGF stimulation (Supplementary Fig. S3 A and C), while Spry2 overexpression had the opposite effects on cell cycle progression and apoptosis in MIA PaCa-2 cells (Supplementary Fig. S3 B and D).

**Fig. 6:**
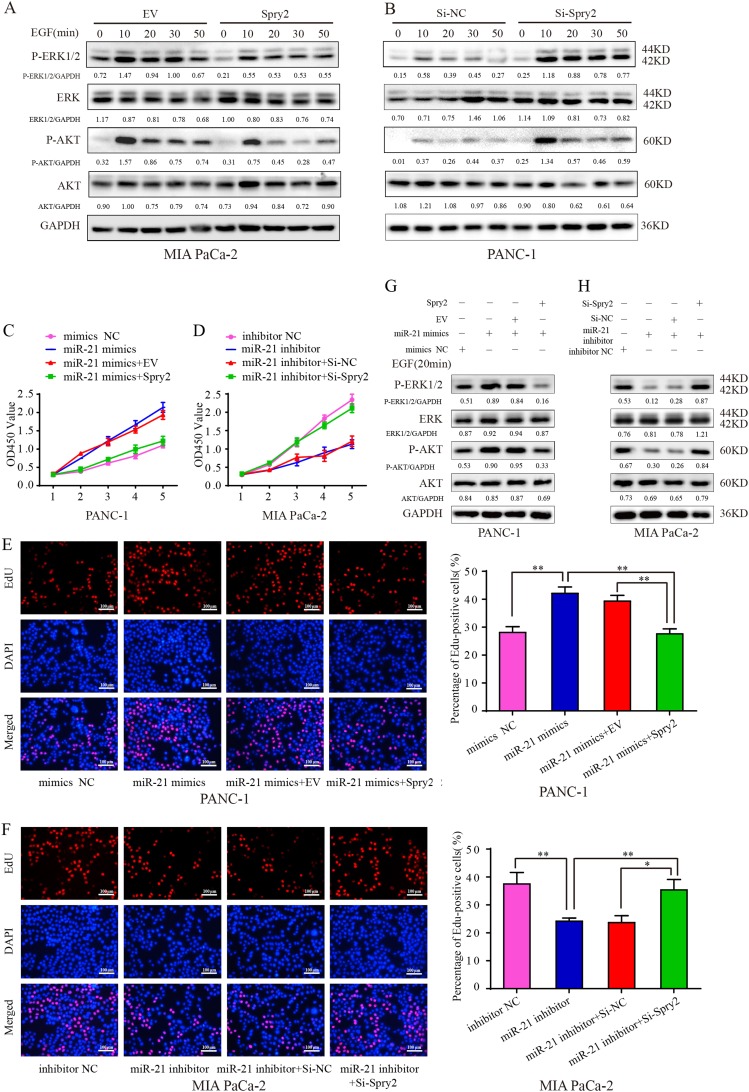
Spry2 mediates the effects of miR-21 on pancreatic cancer cells. **a** After being serum-starved for 24 h, MIA PaCa-2 cells transfected with EV or Spry2 plasmid were incubated in the absence or presence of 50 ng/ml EGF for the indicated times. Western blotting analysis of the indicated proteins was then carried out. Western blotting expression densitometry data was quantified by imageJ and then was normalized to the corresponding densitometry of GAPDH. **b** After being serum-starved for 24 h, PANC-1 cells were transfected with control siRNA (si-NC) or siRNA targeting Spry2 (si-Spry2) and incubated in the absence or presence of 50 ng/ml EGF for the indicated times. Western blotting analysis of the indicated proteins was then carried out. Western blotting expression densitometry data was quantified by imageJ and then was normalized to the corresponding densitometry of GAPDH. **c** miR-21 overexpressing PANC-1 cells transfected with EV or Spry2 overexpression plasmids were incubated in the presence of 50 ng/ml EGF. Then, CCK-8 proliferation assay was used to analyze the growth of PANC-1 cells. **d** miR-21 knockdown MIA PaCa-2 cells transfected with si-NC or si-Spry2 were incubated in the presence of 50 ng/ml EGF. Then, CCK-8 proliferation assay analyzed the growth of MIA PaCa-2 cells. **e**, **f** EdU assay further confirmed the proliferative activity of PANC-1 cells and MIA PaCa-2 cells. **g** Spry2 overexpression weakened the phosphorylation level of indicated proteins in miR-21 overexpressing PANC-1 cells after 50 ng/ml EGF treatment for 20 min. Western blotting expression densitometry data was quantified by imageJ and then was normalized to the corresponding densitometry of GAPDH. (H)Spry2 knockdown amplified the phosphorylation level of indicated proteins in miR-21 knockdown MIA PaCa-2 cells after 50 ng/ml EGF treatment for 20 min. Western blotting expression densitometry data was quantified by imageJ and then was normalized to the corresponding densitometry of GAPDH. Three independent experiments were performed for each group. All data are represented as the mean ± SD. *P < 0.05; **P < 0.01; ***P < 0.001

Prolonged EGF signaling due to lack of negative feedback regulators may cause deregulated cell growth and proliferation. Spry genes, which encode intracellular antagonists of RTK signaling, could impact both the MAPK/ERK and PI3K/AKT pathways through interaction with GRB2, which is a SH2/SH3 adapter protein that linked to RTKs activation in both signaling pathways^23^. Then, we conducted a series of experiments to assess whether Spry2 alone was sufficient to modulate EGF signaling in pancreatic cancer cells. In our study, Spry2 overexpression attenuated EGF-induced ERK1/2 and AKT phosphorylation in MIA PaCa-2 cells (Fig. 6a); meanwhile, in the PANC-1 cells treated with si-Spry2, the degree and duration of EGF-induced ERK1/2 and AKT phosphorylation were stronger than those in control group cells (Fig. 6b).

To further confirm that Spry2 is a functional mediator of miR-21, miR-21 overexpressing PANC-1 cells were transfected with Spry2 overexpression plasmids or empty plasmids. We found that following Spry2 overexpression, miR-21 was no longer able to enhance EGF-induced cancer cell proliferation (Fig. 6c, e) or phosphorylation of ERK1/2 and AKT (Fig. 6g). Similarly, miR-21 suppression in MIA PaCa-2 cells dampened EGF-induced proliferation and signaling pathway activation, which could be rescued by transfection with si-Spry2 (Fig. 6d, f, h). These data confirmed that miR-21 promoted pancreatic cancer cell responses to EGF by suppressing Spry2.

### Clinical pathological features of miR-21 and Spry2 in PDAC patients

**Fig. 7:**
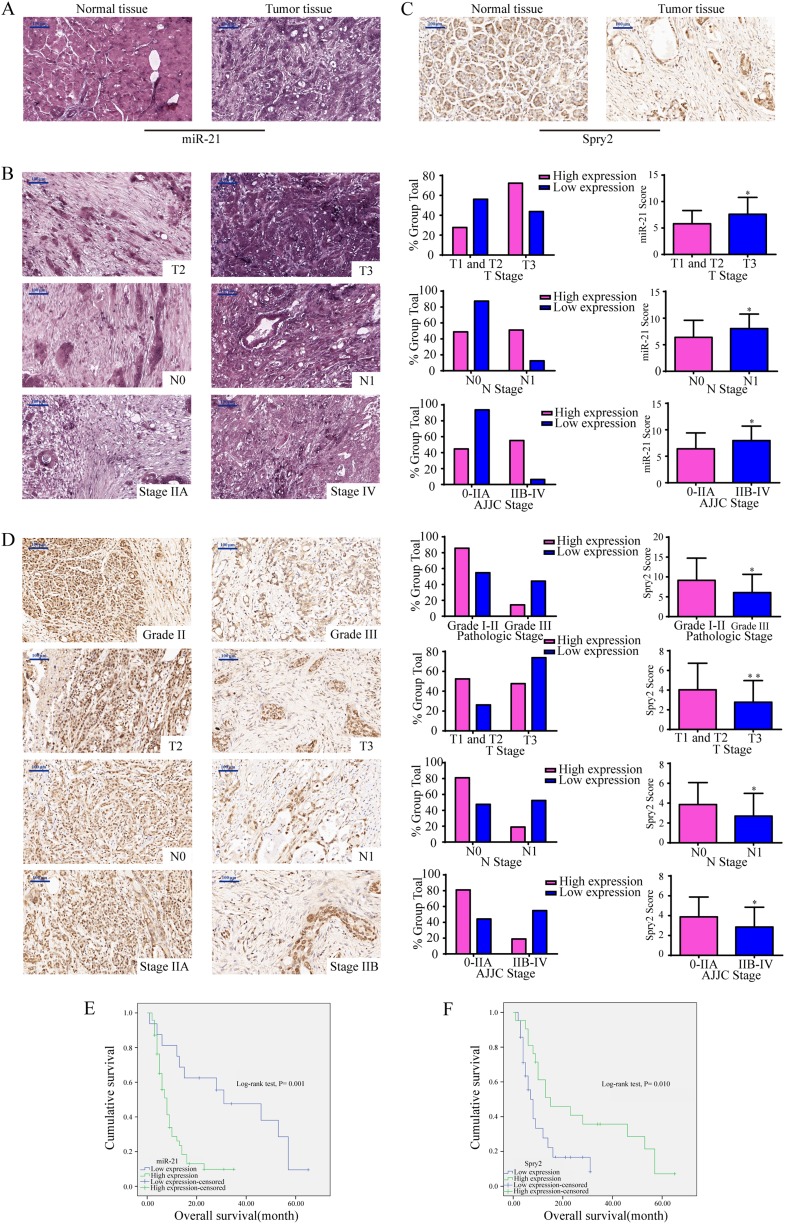
Both miR-21 and Spry2 were closely correlated with clinical pathological features in PDAC patients. **a** Representative images of the miR-21 level in normal pancreatic tissues and matched pancreatic cancer tissues. **b** miR-21 expression in T1 and T2 *vs* T3, N0 *vs* N1, and AJJC stage 0-IIA *vs* IIB-IV. **c** Representative images of Spry2 expression in normal pancreatic tissues and matched pancreatic cancer tissues. **d** Spry2 expression in grade I-II *vs* grade III, T1 and T2 *vs* T3, N0 *vs* N1, AJJC stage 0-IIA *vs* IIB-IV. **e** The OS of patients with high miR-21 *vs* low miR-21 expression. **f** The OS of patients with high Spry2 *vs* low Spry2 expression. All data are represented as the mean ± SD. *P <0.05; **P < 0.01

First, the levels of miR-21 and Spry2 protein were detected in pancreatic TMAs. We found that miR-21 expression level was significantly higher in tumor tissues than in adjacent normal tissues (Fig. 7a). Further analyses demonstrated that high miR-21 expression significantly affected the T stage, N stage and AJCC stage (*P* < 0.05 for all, Supplementary Table 1 and Fig. 7b). Consistent with the function of Spry2 detected in our study, Spry2 protein expression was low in pancreatic cancer tissues (Fig. 7c), and downregulated Spry2 expression was associated with pathological grade, T stage, N stage and AJCC stage (*P* < 0.05 for all, Supplementary Table 2 and Fig. 7d). Furthermore, Kaplan-Meier survival curves along with the log-rank test demonstrated that high miR-21 expression predicted worse overall survival (OS) in PDAC patients (*P* < 0.05, Fig. 7e), and that low expression of Spry2 also notably reduced OS (*P* < 0.05, Fig. 7f). These data demonstrated that high levels of miR-21 and decreased Spry2 expression played important roles in PDAC progression and could be indicators of poor survival in PDAC patients.

## Discussion

miR-21, one of the most studied miRNAs, is closely related to poor prognosis, including in PDAC^24^. Numerous experiments have demonstrated that miR-21, as an oncogene in PDAC^25–27^, is upregulated not only in PDAC but also in early pancreatic precursor lesions compared with normal pancreas^16^. The reasons for the increase of miR-21 remain unknown. Intriguingly, the mechanism underlying miR-21 overexpression is related to Ras or EGFR in different cell lines. miR-21, as both a target and a regulator of AP-1, can be induced by AP-1 in response to RAS^28^. In PDAC, miR-21 overexpression is an early event in the progression of PDAC precursor lesions and both KRAS (G12D) and EGFR could promote miR-21 production^16^. As shown by research in lung carcinogenesis, EGFR can increase the expression of miR-21. Targeting miR-21 may improve the therapeutic effect of EGFR tyrosine kinase inhibitors (EGFR-TKIs) in lung cancer^29^. HER2/neu, an oncogenic RTK, by initiating MAPK/ERK1/2 pathway, could induce the expression of miR-21, which then increased breast cancer cells invasion and metastasis^30^. In our study, we demonstrated that EGF stimulation could increase miR-21 level, which in turn promoted EGF-induced cell proliferation. Next, we found that miR-21 affected PDAC cell proliferation and apoptosis by targeting the MAPK/ERK and PI3K/AKT signaling pathways, two main downstream pathways of EGF. We concluded that there may be a positive feedback loop in PDAC between miR-21 and the EGF signaling cascade in which miR-21 represses EGF-suppressing factors to increase EGF activity, which in turn promotes miR-21 transcription. Taken together, these findings confirm the carcinogenic effect of miR-21in PDAC.

It is well known that miRNAs bind to perfect or imperfect base sequences, leading to the degradation of targeted mRNAs or suppression of their translation^6,31^. Hundreds of mRNAs are predicted as possible targets of miR-21 by computational algorithms^32,33^; however, few have been experimentally validated. Of the potential targets, Spry2 stimulates our interest the most. Known as a negative regulator of RKT signaling pathways, Spry2 has been shown to regulate cancer cell behaviors, such as cell proliferation and survival and act as a tumor suppressor^21,22^. The expression of Spry2 protein is frequently downregulated in human hepatocellular carcinoma (HCC)^34,35^ and loss of Spry2 significantly induces MAPK activation and promotes the development of liver lesions^36^. A recent study on chronic lymphocytic leukemia (CLL) also showed that Spry2 expression was significantly decreased in CLL cells and had a negative correlation with the prognosis. Furthermore, molecular mechanism studies indicate that Spry2 acted as a negative regulator of BCR and MAPK signaling to facilitate CLL development^37^. In our study, we confirmed the negative correlation between miR-21 and Spry2 expression levels in both PDAC cells and tissues. Moreover, we further verified that Spry2, as a direct target of miR-21, could mediated the effects of miR-21 on pancreatic cancer cell proliferation by inhibiting MAPK/ERK and PI3K/AKT signal pathway.

The MAPK pathways are known to regulate cell proliferation, differentiation and survival in response to extracellular signals^38^. As members of MAPK family, ERK1/2 pathway is often activated by growth factors and considered as prosurvival and oncogenic. The MAPK/ERK1/2 was proved to stimulate cell proliferation indirectly by enhancing AP-1 activity^39^. ERK1/2 also controls cell survival by protecting cells from apoptosis. In cerebellar granular cells, ERK1/2 could inactivate the pro-apoptotic protein BAD to prevent apoptosis^40^. The other key mechanism for regulating pancreatic cancer cell proliferation, survival, and metabolism is the PI3K/AKT pathway. Previous study demonstrated that PI3K/AKT signal pathway increased C-myc and CyclinD1 expression, thereby promoting cell proliferation and cell-cycle G1/S transition^41^. Besides promoting cell proliferation, PI3K/AKT pathway also could block Bad-mediated cell death by phosphorylating Bad^42^. Here, we showed that both miR-21 and Spry2 could affect MAPK/ERK and PI3K/AKT signal pathway, which further explains the role of miR-21 and Spry2 in pancreatic cancer cell proliferation.

In summary, miR-21 not only regulates the EGF signaling pathway by targeting Spry2, but also is responsive to EGF signaling, thus constituting a self-enhancing circuit of the pathway. Our study shows that miR-21 could enhance EGF-induced proliferation through regulation of Spry2 and subsequent activation of MAPK/ERK and PI3K/AKT signaling pathways. In addition, ISH and IHC results reveal that overexpression of miR-21 and decreased expression of Spry2 are associated with adverse clinical features of PDAC patients. Consequently, our results suggest that miR-21 and its target Spry2 play an important role in EGF-induced cell proliferation, which may be helpful to provide new therapeutic target for PDAC patients in the future.

## Electronic supplementary material

Supplementary Figure S1

Supplementary Figure S2

Supplementary Figure S3

Supplementary legend marked

Supplementary legend

STR Profiling Report PANC-1

STR Profiling Report MIAPACA-2
