# Supplementary figures and images for: miR-21 promotes EGF-induced pancreatic cancer cell proliferation by targeting Spry2
Source: Cell Death Dis. 2018 Nov 21;9(12):1157. doi: 10.1038/s41419-018-1182-9 (PMC6249286; doi:10.1038/s41419-018-1182-9)

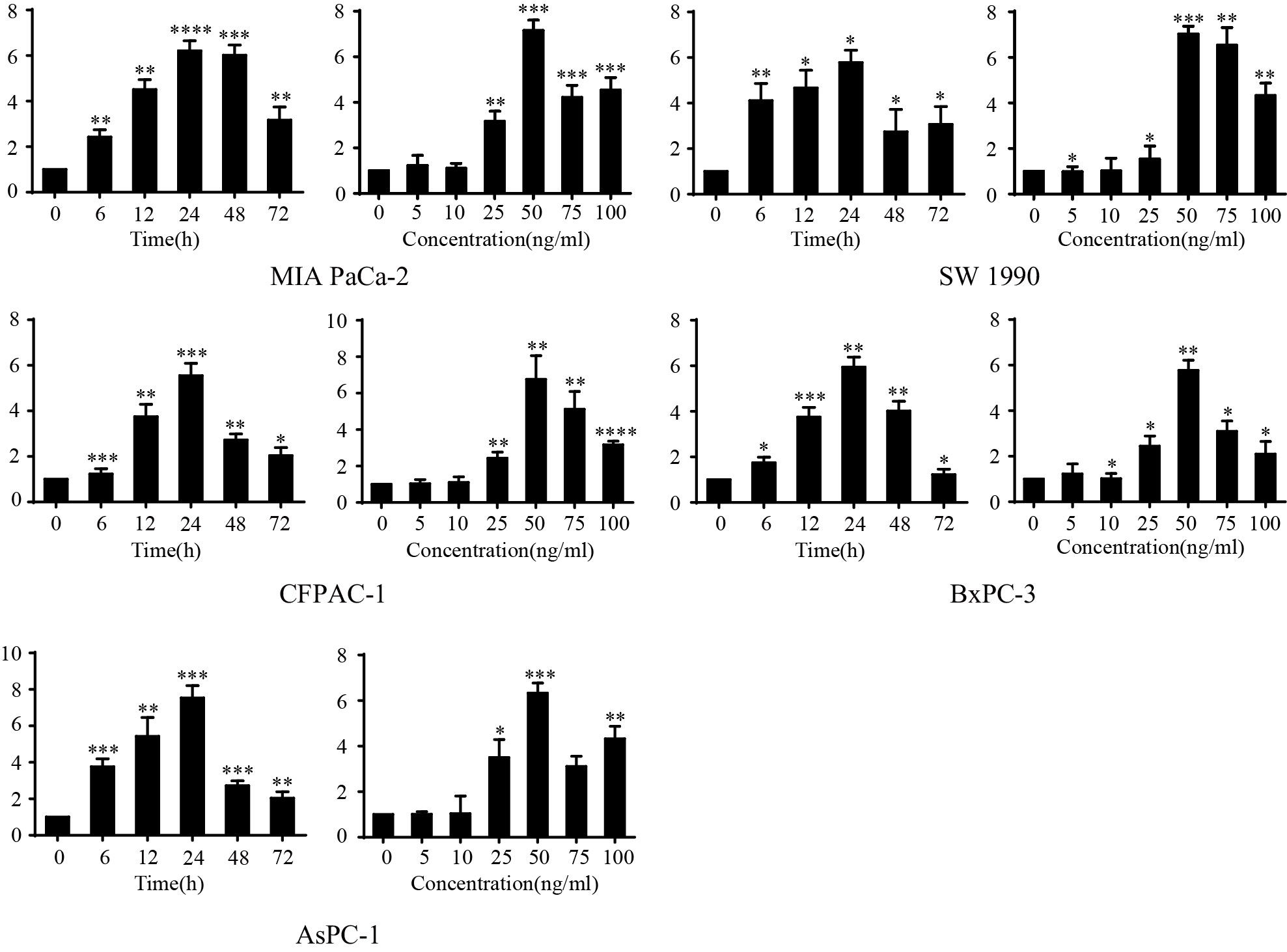

Supplement: Supplementary file 1 — Supplementary Figure S1 [file 41419_2018_1182_MOESM1_ESM.jpg]

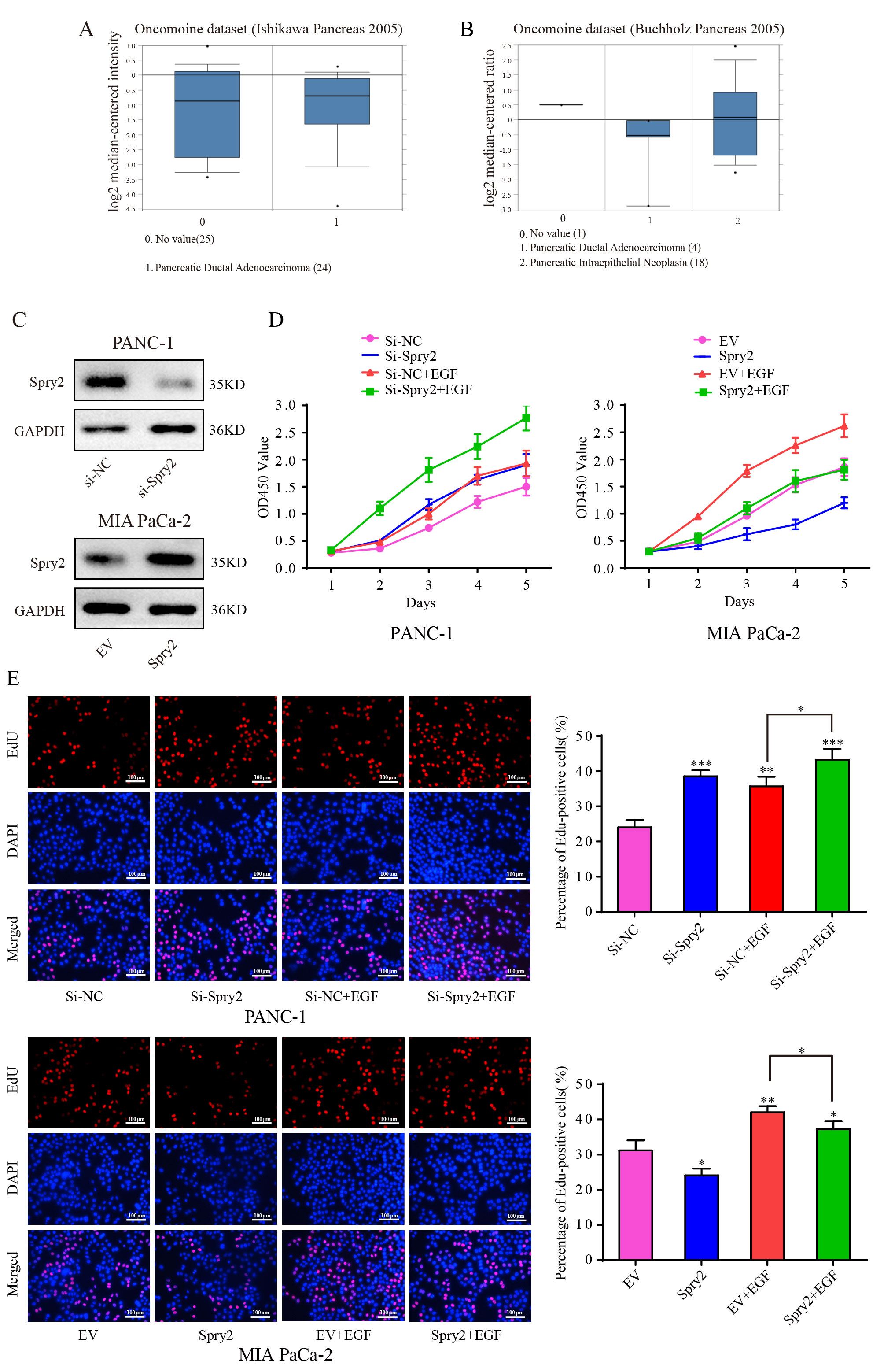

Supplement: Supplementary file 2 — Supplementary Figure S2 [file 41419_2018_1182_MOESM2_ESM.jpg]

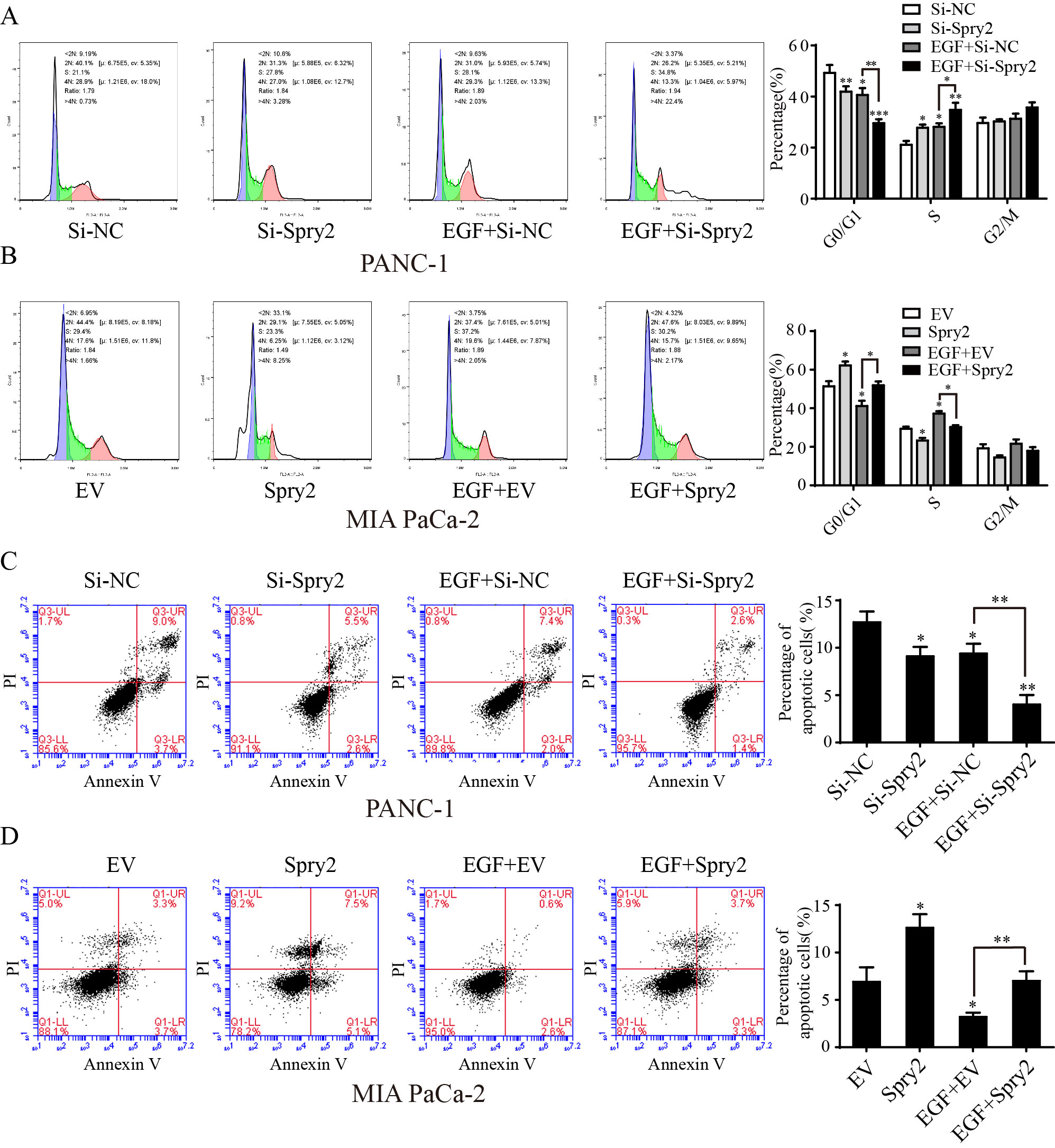

Supplement: Supplementary file 3 — Supplementary Figure S3 [file 41419_2018_1182_MOESM3_ESM.jpg]
