# Supplementary material for: miR-21 promotes EGF-induced pancreatic cancer cell proliferation by targeting Spry2
Source: Cell Death Dis. 2018 Nov 21;9(12):1157. doi: 10.1038/s41419-018-1182-9 (PMC6249286; doi:10.1038/s41419-018-1182-9)
Supplement: Supplementary file 4 — Supplementary legend marked [file 41419_2018_1182_MOESM4_ESM.docx]

**Supplementary figure legend:**

**Supplementary Figure S1**

The concentration and time-dependent effect of EGF on miR-21 level in other pancreatic cancer cell lines. Three independent experiments were performed for each group. All data are represented as the mean±SD. *P＜0.05, **P<0.01, ***P<0.001，****P<0.0001.**Supplementary Figure S2**

**Spry2 alteration mimics miR-21 in EGF-induced pancreatic cancer cell growth.** (A, B) The Oncomine database showed that Spry2 mRNA expression was decreased in pancreatic adenocarcinoma (Ishikawa Pancreas 2005, Buchholz Pancreas 2005). (C) Western blotting was conducted to measure the level of Spry2 protein in PANC-1 and MIA PaCa-2 cells. (D) Growth of PANC-1 cells and MIA PaCa-2 cells were measured by CCK8 proliferation assay. (E) EdU assay further analysis the proliferation of PANC-1 cells and MIA PaCa-2 cells. Three independent experiments were performed for each group. All data are shown as the mean±SD. *P＜0.05, **P< 0.01, ***P<0.001.

**Supplementary Figure S3**

(A, B) Flow cytometry analysis of cell cycle of PANC-1 cells and MIA PaCa-2 cells

(C, D) Flow cytometry analysis of cell apoptosis in PANC-1 cells and MIA PaCa-2 cells. Three independent experiments were performed for each group. All data are shown as the mean±SD. *P＜0.05, **P< 0.01, ***P<0.001.
