# Supplementary material for: miR-21 promotes EGF-induced pancreatic cancer cell proliferation by targeting Spry2
Source: Cell Death Dis. 2018 Nov 21;9(12):1157. doi: 10.1038/s41419-018-1182-9 (PMC6249286; doi:10.1038/s41419-018-1182-9)
Supplement: Supplementary file 6 — STR Profiling Report PANC-1 [file 41419_2018_1182_MOESM6_ESM.pdf]

SHANGHAI BIOWING BIOTECHNOLOGY Co. LTD
Your Intimate Partner in Bioscience!

Cell Line Authentication Service

STR Profiling Report

Sample Type: Cell Line

Sample from: Shanghai General Hospital, Shanghai Jiao Tong University School of Medicine

Testing Method: STR Genotyping

Report Time: July 27, 2017

COMPANY STATEMENT

1. THIS REPORT IS ONLY RESPONSIBLE FOR THE SAMPLES ANALYZED.
2. THE TESTING RESULTS AND THE ORGANIZATION NAME WILL NOT BE USED FOR ADVERTISEMENT, COMMERCIAL EXHIBITIONS, COMMERCIAL PERFORMANCE AND OTHER COMMERCIAL ACTIVITIES.
3. OBJECTIONS SHOULD BE RAISED WITHIN FIFTEEN DAYS AFTER THE RECEIPT OF THIS REPORT.
4. THE PAPER REPORT WITH CONTENT ALTERING, ADDING OR WITHOUT THE STAMPED SEAL OF THE COMPANY ARE INVALID.

Testing Company: Shanghai Biowing Applied Biotechnology Co. Ltd

Address: Room 4F, 8 th Buiding,Guiguo Garden,NO.471 Guiping Road,Caohejing Development Zone,Shanghai

Tel: +86-021-33559491

Contact: YiQun Chen

E-mail: biowing@vip.163.com

Cell Line Authentication – STR Profiling Report

Sample code

Table 1. Sample Code

Customer's code	Company Code
311	20170721-18

Sample Number:1

Sample Type: Cell line

Testing Type: STR

Sample From: Shanghai General Hospital, Shanghai Jiao Tong University School of Medicine

Testing Method:

DNA was extracted by a commercial kit from CORNING (AP-EMN-BL-GDNA-250G). The twenty STRs including Amelogenin locus were amplified by six multiplex PCR and separated on ABI 3730XL Genetic Analyzer. The signals were then analyzed by the software GeneMapper.

Data Interpretation:

Cell lines were authenticated using Short Tandem Repeat (STR) analysis as described in 2012 in ANSI Standard (ASN-0002) by the ATCC Standards Development Organization (SDO) and in Capes-Davis et al., Match criteria for human cell line authentication: Where do we draw the line? Int J Cancer.2013;132(11):2510-9.

Test Results:

1. Result

Table 2. Matching information on the cell lines

Sample Code	Multi-allele	Cell line matched	Cell Bank	EV	Percentage
20170721-18	No	PANC-1	DSMZ	1.00	9/9

- Multi-allele means some STR contain more than two loci.

2. Sample Description

A. The STR results showed that no multiple alleles were found in this cell line, and no cross contamination of human cells was found in the cell line.

B. The DNA of the cell lines found to perfect match the type of cell lines in a cell line retrieval, DSMZ database shows that cells called **PANC-1.** corresponding to the cell number **783.**

EV	Cell No.	Cell name	D5S818	D13S317	D7S820	D16S539	VWA	TH01	AM	TPOX	CSF1PO
	Query (Your Cell)		11, 13	11, 11	8, 10	11, 11	15, 15	7, 8	x, x	8, 11	10, 12
1.00(36/36)	783	PANC-1	11, 13	11, 11	8, 10	11, 11	15, 15	7, 8	X, X	8, 11	10, 12
1.00(36/36)	CRL-1469	PANC-1	11, 13	11, 11	8, 10	11, 11	15, 15	7, 8	X, X	8, 11	10, 12
1.00(36/36)	JCRB0177.0	KP-1N	11, 13	11, 11	8, 10	11, 11	15, 15	7, 8	X, X	8, 11	10, 12
1.00(36/36)	RCB2095	PANC-1	11, 13	11, 11	8, 10	11, 11	15, 15	7, 8	X, X	8, 11	10, 12

3. Genotyping Result

Table 3. STR and Amelogenin Genotyping Results of Cell line 20170721-18

Loci	Sample information			Cell Bank information		
	Sample name：311			Cell line name：PANC-1		
	Allele1	Allele2	Allele3	Allele1	Allele2	Allele3
D5S818	11	13		11	13	
D13S317	11	11		11	11	
D7S820	8	10		8	10	
D16S539	11	11		11	11	
VWA	15	15		15	15	
TH01	7	8		7	8	
AMEL	X	X		X	X	
TPOX	8	11		8	11	
CSF1PO	10	12		10	12	
D12S391	22	22				
FGA	21	21				
D2S1338	23	24				
D21S11	28	28				
D18S51	12	12				
D8S1179	14	15				

D3S1358	17	17	
D6S1043	11	12	
PENTAE	7	14	
D19S433	11	16	
PENTAD	14	14	

Others:

1. Genotyping Strategy and Site Distribution

Attached Table. Experimental Strategy and Sites

	**Strategy 1**	**Strategy 2**	**Strategy 3**	**Strategy 4**
1	TH01	TPOX	D3S1358	AMEL
2	D12S391	VWA	D13S317	D5S818
3	D7S820	D8S1179	D6S1043	D2S1338
4	CSF1PO	PENTAD	D16S539	D21S11
5	FGA		D19S433	D18S51
6	PENTAE			

The allele match algorithm compares the 8 core loci plus amelogenin only, even though alleles from all loci will be reported when available.

2. DSMZ tools was used to carry on the cell line comparison, which contains 2455 cell lines STR data from ATCC, DSMZ, JCRB ,ECACC，GNE and RIKEN databases. If the cell is not included in the above cell library, users need to compared with other databases.

Technician:Menglu Shen
Check: Yang Bai
Person in Charge: Yiqun Chen

Issue date: July 27, 2017
